# The New American Joint Committee on Cancer T staging system for stomach: increased complexity without clear improvement in predictive accuracy for endoscopic ultrasound

**DOI:** 10.1186/s12876-020-01558-8

**Published:** 2021-06-11

**Authors:** Chaoqun Han, Tao Xu, Qin Zhang, Jun Liu, Zhen Ding, Xiaohua Hou

**Affiliations:** 1 Division of Gastroenterology, Union Hospital, Tongji Medical College, Huazhong University of Science and Technology, Wuhan, 430022 China; 2 Department of Pathology, Union Hospital, Tongji Medical College, Huazhong University of Science and Technology, Wuhan, 430022 China

**Keywords:** Endoscopic ultrasound, Gastric cancer, American Joint Committee on Cancer, Staging, Accuracy

## Abstract

**Background:**

The efficacy of endoscopic ultrasound (EUS) for determining the T category of gastric cancer is variable. The aim of this study was to evaluate the superiority of EUS by using the 6th edition American Joint Committee on Cancer (AJCC) staging system for stomach cancer compared to the new 7th/8th edition.

**Methods:**

A retrospective analysis of clinical and EUS imaging features of 348 gastric carcinoma patients who underwent radical resection were retrospectively analyzed. Differences between the 6th and 7th/8th edition T staging systems for preoperative EUS evaluation were compared.

**Results:**

The accuracy of EUS T staging was 72.4% for the 7th/8th edition and 78.4% for the 6th edition. T3 stage accuracy was significantly worse when the T3 group status was changed. The tumor location, echoendoscope type, and histological type were associated with inaccuracy. We further analyzed the EUS image features for each tumor T stage and found that an indistinctly visible muscularis propria (MP) or with obvious thickening was considered an indicator of lesions involved in the MP with a sensitivity of 81.3%; an MP completely disappeared and accompanied with a serosal layer intact may be a marker that the lesion invaded to the subserosa. We also found that irregularities in the outer edge of the gastric wall were markers of gastric serosal layer penetration with a positive predictive value of 92.2%.

**Conclusions:**

The increased complexity of the 7th/8th edition T staging system is accompanied by worsening of the predictive accuracy for EUS as compared to the 6th edition. Furthermore, the tumor location, echoendoscope type, histological type, and EUS image features for each tumor T stage should warrant attention.

## Background

Gastric cancer is the fourth most common malignancy and the second leading cause of cancer-related deaths worldwide [1]. It also has a poor prognosis and high mortality [2–4]. Accurate categorization of the tumor stage, including the invasive depth and lymph node status, is crucial for prognostic assessment and initial therapeutic decisions for patients.

Currently, the tumor-lymph node-metastasis (TNM) staging system based on the anatomic extent of malignancies has been a benchmark for prognosis evaluation. In order to maintain the relevancy of the staging system, the American Joint Committee on Cancer (AJCC) and International Union Against Cancer have collaborated on aperiodic revisions of this staging system, leading to the 6th edition in 2002, the 7th edition in 2010 [5], and the 8th edition in 2017 [6, 7].

For gastric cancer, several changes were made to the 6th edition. In this new edition, the T classification categories have been redefined. According to the 7th/8th edition, the 6th edition T2b classification was redefined as T3 (subserosa) and the T3 stage was classified as T4a (serosa). With these complicated revisions, there are improvements in the prognostic value in terms of homogeneity, discriminatory ability, and monotonicity of gradients for patients with gastric cancer [3, 8].

However, accurate preoperative staging is important for selecting the most effective treatment. Endoscopic ultrasonography (EUS) provides the ability to differentiate anatomic structural layers of the gastric wall and show remarkable differences in their echogenic appearance if a tumor has invaded [9, 10]. EUS is considered the best imaging modality for local and regional staging of gastric cancer compared to other methods, especially for determining the T category [11–14]. Although the 7th/8th edition TNM staging system provides a more detailed classification, they seem to be more complex to apply than the 6th edition. In our study, we aimed to evaluate the validity of EUS comparing the 6th edition to the 7th/8th edition of the AJCC T staging system for gastric cancer. Furthermore, we attempted to identify the sonographic features that affect the accuracy of EUS staging.

## Methods

### Patient selection

A total of 1044 patients with gastric cancer treated at our center from December 2015 to December 2018 were included. To obtain the correct histological staging, all patients included in this analysis met the following criteria: (1) disease was pathologically defined as gastric cancer; (2) tumor-free resection (R0) margin status; and (3) pre-operative staging by EUS. The exclusion criteria were: (1) surgery preceded by preoperative chemoradiotherapy (neoadjuvant therapy) or palliative surgery; (2) patients with distant metastasis; (3) patients with synchronous malignancies or previous other primary cancers; and (4) patients with bulky obstructions that EUS failed to pass through. The study was approved by the Ethics Committee of Tongji Medical College, Huazhong University of Science and Technology (No: IORG0003571). All patients provided written informed consent for the EUS operation and data were anonymized and de-identified.

### EUS equipment and technique procedures

A 360° radial echoendoscope (Olympus processor EU-ME2, Olympus, Tokyo, Japan) was used with de-aerated water (300–800 mL) or fitted with a water-filled balloon to assist acoustic coupling and improve the transmission of the ultrasound beam with variable frequencies of 7.5, 10, and 12 Hz. Analogous to pathologic classification, the extent of wall invasion was imaged as a hypoechoic disruption and evaluated based on the tumor infiltration into each layer [15]. All patients underwent assessment of the tumor invasion depth by EUS and were restaged using the 6th, 7th, and 8th editions of the AJCC TNM staging system [16, 17]. The three different T stage versions are shown in Table 1. All operations were performed by an experienced gastroenterologist with experience of more than 1000 EUS per year.

**Table 1:** Changes in the AJCC T staging system for gastric cancer

Tumor	AJCC 6th ed.	AJCC7th ed.	AJCC8th ed.
T: Primary tumor			
Depth of invasion			
No evidence of primary tumor	T0	T0	T0
Carcinoma-in-situ	Tis	Tis	Tis
Lamina propria or muscularis mucosa	T1	T1	T1
Or submucosa	T1	T1	T1
Muscularis propria	T2	T2	T2
Subserosa	T2	T3	T3
Serosa	T3	T4	T4
Adjacent structures	T4	T4	T4

For AJCC 6th edition: T1: tumor invasion limited to the mucosal or submucosal layer; T2: destruction of the muscularis propria or subserosal layer (T3 for AJCC 7th/8th edition); T3: cancer penetrating the serosa ((T4 for AJCC 7th/8th edition)); and T4: disease invasion in the vicinity of the stomach or other organs

### Data collection

In order to compare the difference in EUS efficacy between the AJCC 6th and 7th/8th editions, EUS accuracy was determined by comparing the tumor depth on EUS that on pathology. Two investigators who were blinded to patients’ names and reports, independently evaluated the restaging results and verified reciprocally. Any discrepancies in opinion were resolved by discussion, or adjudicated by a third reviewer. We also focused on the factors that may have influenced the accurate diagnosis of EUS tumor invasion depth. The factors included patient demographics (age, gender), clinicopathologic details (lesion situation, histological classification), and ultrasonic characteristics (lesion size, ascites, EUS type). The histological classification used in this study was depended on the WHO 2019 classification. A challenge in the identification of nodes with EUS is the inability to visualize nodes that are outside the range of the transducer [18–20]. Thus, gastric cancer N staging remains an area of uncertainty. The efficacy of EUS N staging and other related data are not shown.

### Statistical analysis

All patients were restaged using the 6th and 7th/8th editions of the AJCC T staging system. For statistical analysis, categorical variable results are presented as numbers and percentages, and continuous variables are presented as the mean ± standard deviation (SD). The possible influence of categorical or non-categorical factors was conducted using Pearson’s chi-squared tests and t-tests. Subsequently, logistic regression models were performed to assess the potential associations related to EUS accuracy. Statistical analysis was performed using IBM SPSS Statistics software (version 20.0, IBM Corp, Armonk, NY, USA). A significance level of *P* ≤ 0.05 was used for all models (two-sided).

## Results

### General patient characteristics

In total, 348 patients were included in this study. The mean patient age was 56.7 years (range, 29–77 years), and 61.2% were male. With regards to the location of lesions, the main tumor occurrence was located in the antrum and corpus (64.4%) and T3 staging accounted for the majority of cases. Tumors were well, moderately, and poorly differentiated in 11.2, 13.8, and 47.4% of cases, and signet ring cell adenocarcinoma was observed in 27.6%. We also identified EUS image characteristics, including the presence of circumferential lesions (cancer extension beyond a semi-circular area, 34.5% of tumors were circumferential lesions≥1/2), and ascites (8.6%). The clinicopathological characteristics are summarized in Table 2.

**Table 2:** The Basic clinicopathological characteristics of 348 gastric cancer patients

Characteristic	No. of patients (%)
Age (year)	
Mean ± SD	56.7 ± 10.8
Median (P25, P75)	58.0 (50.0,65,0)
Gender	
Male	213 (61.2%)
Female	135 (38.8%)
Longitudinal portions	
Antrum	183 (38.9%)
Corpus	120 (25.5%)
Gastroesophageal junction	63 (13.4%)
Fundus	72 (15.2%)
Gastric angulus	33 (7.0%)
Cross-sectional portions	
Circumferential lesions ≥1/2	120 (34.5%)
Circumferential lesions, < 1/2	228 (65.5%)
Ascites	30 (8.6%)
Absence of ascites	318 (91.4%)
EUS type	
Radial scanning	309 (88.8%)
Linear array	39 (11.2%)
Histological type	
Well-differentiated	39 (11.2%)
Moderately differentiated	48 (13.8%)
Poorly differentiated	165 (47.4%)
Signet ring cell adenocarcinoma	96 (27.6%)
6th AJCC pathologic T category	
pT1	45 (13.0%)
pT2	123 (35.3%)
pT3	174 (50.0%)
pT4	6 (1.7%)
7th/8th AJCC pathologic T category	
pT1	45 (13.0%)
pT2	24 (6.9%)
pT3	99 (28.4%)
pT4	180 (51.7%)

The total of numbers were more than 348 patients in longitudinal portions because some patients have two or more lesions; For histological type, a patient may have two, such as moderately and poorly differentiated types, the worse was for the final result

*SD* standard deviation, *AJCC* American Joint Committee on Cancer, *pT* pathological T stage

### Surgical and pathological results

Table 2 shows the detailed classifications based on the 6th and 7th/8th editions of the TNM classification. Among the major revisions in the 7th/8th edition T classification was that the definition of the T3 and T4 stages were changed. According to the 6th edition, T1, T2, T3, and T4 were seen in 45 (13.0%), 123 (35.3%), 174 (50.0%), and 6 (1.7%) cases, respectively. However, T2, T3 and T4 were seen in 24 (6.9%), 99 (28.4%), and 180 (51.7%) cases, respectively, based on the 7th/8th edition staging systems. Among the 348 patients, the major changes were an increased number of T4 classifications and less T2 and T3 classifications according to the AJCC 7th/8th edition. The redefined T2, T3, and T4 classifications may be more evenly distributed.

### Efficacy of EUS in classifying T stage

Compared with the pT category, the overall accuracy of EUS for T staging was 72.4 and 78.4% for the 7th/8th and 6th edition staging systems, respectively. Detailed comparisons between uT and pT categories are presented in Table 3. Furthermore, the differences in accuracy for the four stages were statistically significant (*P* = 0.001 for the 7th/8th edition and P = 0.001 for the 6th edition). Overall, the frequency of overstaging by EUS was nearly equal to that of understaging (14.7% vs. 12.9%).

**Table 3:** Comparison of EUS-T (uT) categories and pathologic T (pT) categories

uT categories Total		pT categories (AJCC 7th/8th)						
		T1	T2	T3	T4	Correct, %	Overstage,%	Understage, %
T1	42	36	6	0	0	80.0	20.0	0
T2	48	9	12	21	6	50.0	25.5	25.5
T3	54	0	0	42	12	42.4	36.4	21.2
T4	204	0	6	36	162	90.0	0	10.0
pTtotal	348	45	24	99	180	72.4	14.7	12.9
uT categories Total		pT categories (AJCC 6th)						
		T1	T2	T3	T4	Correct, %	Overstage, %	Understage, %
T1	42	36	6	0	0	80.0	20.0	0
T2	102	9	75	18	0	61.0	34.1	4.9
T3	195	0	39	156	0	89.7	0	10.3
T4	9	0	3	0	6	100.0	0	0
pTtotal	348	45	123	174	6	78.4	14.7	6.9

*EUS* endoscopic ultrasonography, *AJCC* American Joint Committee on Cancer

For the AJCC 7th/8th edition, EUS had the highest accuracy in pT4 patients. However, 36.4% of pT3 patients were overstaged as having uT4 lesions by EUS. In pT2 cases, 50.0% were accurately classified, but as many as 25.5% were understaged as uT1 lesions by EUS. For the AJCC 6th edition, EUS also had the highest accuracy in pT4 patients. Unexpectedly, the accuracy of EUS in classifying the T3 category was up to 89.7, and 10.3% of uT2 patients identified by EUS were under-staged pT3 cases. It is also interesting to note that nearly two-thirds (61.0%) of pT2 patients were accurately diagnose. The accuracy of EUS for the T2 and T3 stages was obviously improved based on the AJCC 6th edition.

With regard to T1 cases, our data showed that EUS had a relatively satisfactory accuracy rate. However, it should also be noted that 20% of pT1 patients could actually be assessed by minimally invasive endoscopic resection.

### Factors influencing the staging of gastric cancer by EUS

Among the patients included in the study, the EUS accuracy was not influenced by the size of the gastric lesion or presence of ascites. Interestingly, EUS had the highest accuracy for the corpus (86.7%), and tended to decline in lesions located in the upper third of the stomach (Table 4). Multivariate logistic regression analysis showed that the corpus, radial scanning, and well-differentiated tumors were associated with a higher accuracy of EUS (*P* < 0.05). The gastric angulus appeared to have significant overstaging (*P* = 0.001). The fundus and gastroesophageal junction had a greater possibility of understaging (*P* = 0.014). Further multivariate logistic regression analysis indicated that lesions located in the gastric angulus presented a significantly higher risk of overstaging (*P* = 0.018, odds ratio [OR] = 2.278; Table 5). The accuracy of EUS was also influenced by the echoendoscope type. The linear array presented significantly incorrect staging (*P* = 0.012) and had a greater possibility of understaging (*P* = 0.009). There were also significant differences in the accuracy among the histological types (*P* = 0.039). For well-differentiated tumors, EUS had better staging accuracy relative to that of signet ring cell carcinoma (84.6% vs. 62.5%). Furthermore, when subjected to multivariate analysis, lesions with signet ring cell adenocarcinoma presented significant risk factors for inaccuracy (2.684-fold OR, *P* = 0.001) and understaging (4.800-fold OR, *P* = 0.005).

**Table 4:** Factors affecting EUS T staging accuracy, overstaged and understaged according to clinicopathologic and endoscopic variables

Variables	No.of accuracy (%)	*P*	No. of overstaged (%)	*P*	No. of understaged (%)	*P*
Longitudinal portions		*0.001*		*0.001*		*0.014*
Antrum	129/183 (70.5%)		35/183 (19.1%)		19/183 (10.4%)	
Corpus	104/120 (86.7%)		6/120 (5%)		10/120 (8.3%)	
Gastroesophageal junction	42/63 (66.7%)		8/63 (12.7%)		13/63 (20.6%)	
Fundus	46/72 (63.9%)		10/72 (13.9%)		16/72 (22.2%)	
Gastric angulus	20/33 (60.6%)		10/33 (30.3%)		3/33 (9.1%)	
Cross-sectional portions		0.802		0.202		0.067
Circumferential lesions ≥1/2	88/120 (73.4%)		22/120 (18.3%)		10/120 (8.3%)	
Circumferential lesions < 1/2	164/228 (71.9%)		29/228 (12.7%)		35/228 (15.4%)	
		0.674		0.786		0.399
Ascites	23/30 (76.7%)		5/30 (16.7%)		2/30 (6.6%)	
Absence of ascites	229/318 (72.0%)		46/318 (14.5%)		43/318 (13.5%)	
EUS type		*0.012*		0.481		*0.009*
Radial scanning	231/309 (74.8%)		44/309 (14.2%)		34/309 (11.0%)	
Linear array	21/39 (53.9%)		7/39 (17.9%)		11/39 (28.2%)	
Histological type		*0.039*		0.361		*0.000*
Well-differentiated	33/39 (84.6%)		4/39 (10.3%)		2/39 (5.1%)	
Moderately differentiated	37/48 (77.1%)		6/48 (12.5%)		5/48 (10.4%)	
Poorly differentiated	122/165 (73.9%)		30/165 (18.2%)		13/165 (7.9%)	
Signet ring cell adenocarcinoma	60/96 (62.5%)		11/96 (11.5%)		25/96 (26.0%)

**Table 5:** Multivariate analysis of clinicopathologic and endoscopic factors affecting EUS T staging

Variables	*P*	Odds ratio (95% CI)
Accuracy		
Corpus	0.012	0.451 (0.292–0.886)
Gastric angulus	0.006	2.974 (1.370–6.460)
Radial scanning	0.023	0.773 (0.480–0.962)
Well-differentiated	0.011	0.652 (0.321–0.899)
Signet ring cell adenocarcinoma	0.001	2.682 (1.549–4.933)
Overstaged		
Gastric angulus	0.018	2.278 (1.178–3.544)
Understaged		
Signet ring cell adenocarcinoma	0.005	4.800 (1.264–10.892)

All variables were calculated by binary or multivariate logistic regression analysis. Results for variables with *P* > 0.05 were not shown

*CI* confidence interval; *P* p value

### EUS image features for different tumor T stages

**Fig. 1:**
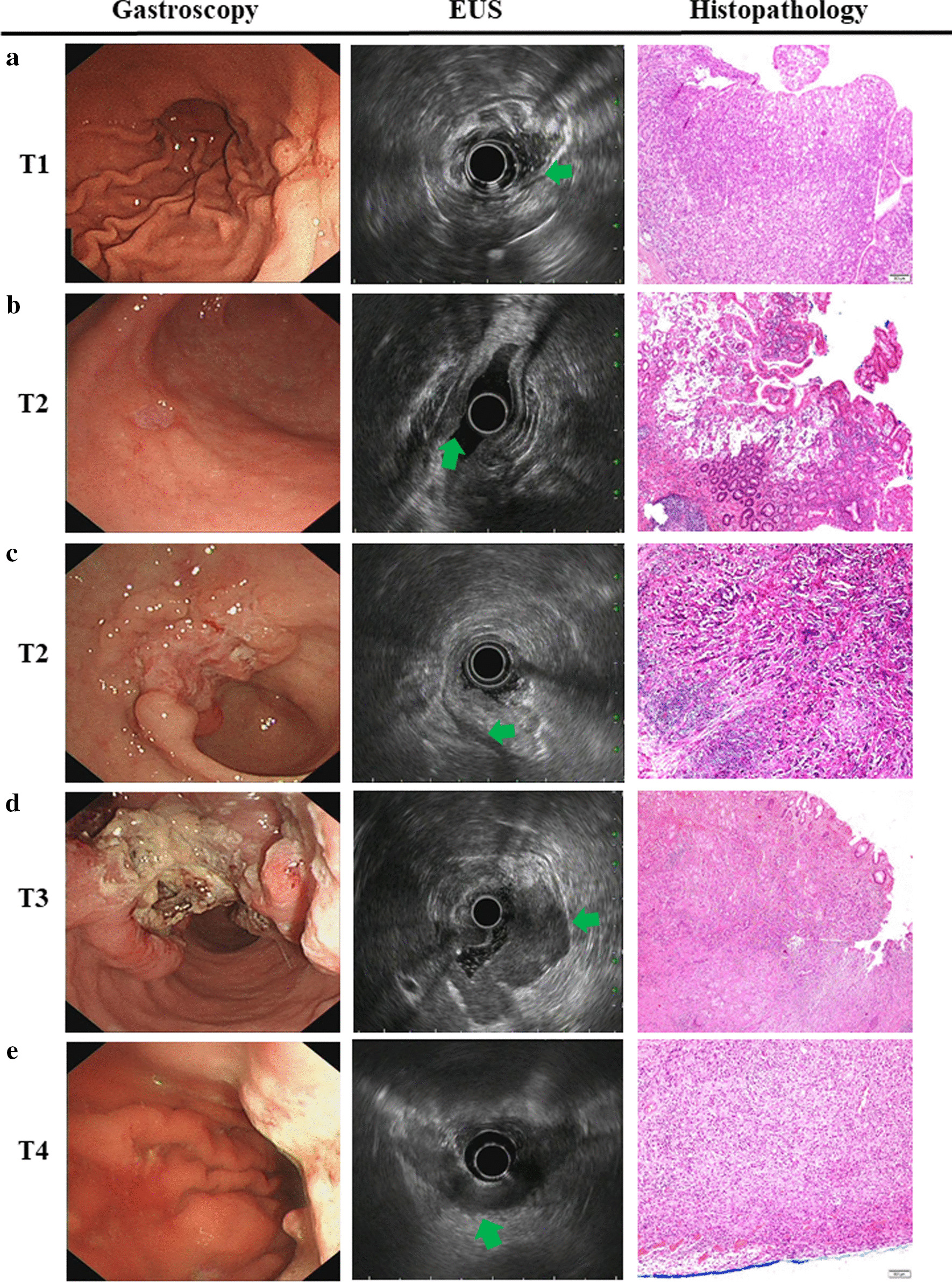
The tumor T stage depend on AJCC 7th/8th edition system and EUS image features for each tumor T stage. **a** Endoscopic image of the lesion showed an ulcer located in the posterior wall of the body with peripheral mucosal consolidation. EUS image showed disappearance of first hyperechoic layer, mild thickness and hypoechoic change of the second hypoechoic layer, and normal third hyperechoic layer (arrows). Surgical resection confirmed poorly-differentiated and partial signet-ring cell gastric cancer confined to submucosal layer; **b** Gastroscopy showed an ulcer located in the anterior wall of gastric angulus. EUS image of the lesion showed disappearance of the first three layers and companied by muscularis propria visible indistinctly (arrows). The surgical specimen confirmed poorly-differentiated gastric cancer confined to the submucosal layer; **c** Endoscopic image showed a neoplasm located in the anterior wall of the antrum. EUS image of the lesion showed disappearance of the first three layers and companied by muscularis propria obvious thickening (arrows). The surgical specimen confirmed tumor infiltrated to the muscularis propria layer; **d** Endoscopic image of the lesion showed a neoplasm located in the lesser curvature side of the antrum with dirty surface. EUS image showed a thick hypoechoic lesion spreading from the mucosal to muscularis propria layer with an intact serosa layer (arrows). The surgical specimen confirmed tumor infiltrated to the subserosa; **e** Endoscopic image showed a large ulcer located in the upper posterior wall of the gastric body. EUS image showed an obviously thick hypoechoic lesion that spread throughout the entire wall and invaded the serosa infiltration (arrows). The serosal layer was irregularities in the outer edge of the gastric wall. The surgical specimen confirmed lesion confined to the serosal layer

We then reread all patient results, analyzed the EUS image feature, and found that the hypoechoic change of the first three layers (the mucosal layer to the submucosal layer) was a feature of the T1 stage (Fig. 1a). The accompaniment of an indistinctly visible or obviously thickened muscularis propria (MP) was considered an indicator of lesions involved in the MP (T2 stage; Fig. 1b-c). The sensitivity between EUS and pathological results for this T2 stage feature was 81.3% with a high positive predictive value (PPV) of 83.3%. Furthermore, when the MP disappeared completely and was accompanied with an intact serosal layer, the lesion was involved in the subserosa (T3 stage; Fig. 1d). The consistency rate between the EUS and pathological results was 75.8%. Finally, we also found that irregularities in the outer edge of the gastric wall were markers of gastric serosal layer penetration (Fig. 1e). The consistency rate between EUS and pathological results for serosal involvement was 82.6%. Sensitivity, specificity, PPV, and negative predictive values (NPV) for this characteristic were 84.9, 70.1, 92.2, and 55.2%, respectively. Gastric wall outer edge irregularities are an effective indicator for confirming serosal extension. Figure 1 depicts the EUS features of each T stage.

## Discussion

Tumor stage is an important component guiding the treatment of gastric cancers. The current study describes the impact of EUS accuracy changes for stomach cancer by comparing the 6th and 7th/8th editions of the T staging system. The key findings of this study are as follows: (1) compared with the 7th/8th edition, the 6th edition T staging system may be more accurate and adaptable for the EUS T stage; (2) the tumor location, echoendoscope type, and histological type were associated with inaccuracy; and (3) the EUS image features of each tumor T stage can guide the judgment of the EUS gastroenterologist.

The updated TNM classification system attempts to determine extent of disease, provide guidance for treatment planning, and predict outcomes [21]. Most authors agree that the increased complexity of the 7th/8th edition system is superior to the 6th edition system, especially in evaluating the prognosis of cancer patients [22–24]; however, the impact of the EUS T stage remains unclear. The first substantial major different characteristic between the AJCC 6th and 7th/8th editions is that the T2 stage in the 6th edition system was sub-classified into T2 and T3 in the 7th/8th edition systems. After revision, the accuracy in classifying the T2 and T3 categories were unsatisfactory, for only 50.0 and 42.4% in our study, respectively. However, when the T3 uT category was defined as T2 by the 6th edition, the results could be clearly distinguished with the accuracy reaching 61.0 and 89.7%, respectively.

The new 7th/8th edition strengthens the role of the subserosa is another different characteristic between these two edition systems. This is the main reason why the accuracy of EUS for AJCC 8th edition become somewhat lower than that for previous AJCC editions. From a technical perspective, distinguishing between subserosal and serosal lesions by EUS is challenging. This is also the main reason for the poor accuracy of the T3 stage. However, we found that complete disappearance of the MP accompanied with an intact serosal layer may be a marker of lesion involvement in the subserosa. The consistency rate was 75.8%. For all this, the challenge of accurate T3 staging remains a frequent issue since this T stage tends to be overstaged. In some areas, such as the lesser curvature regions and fundal of the gastric wall are not entirely covered by the serosa [25], which may result in overstaging.

Next, we also found that an indistinctly visible or obviously thickened MP was a marker of lesion involvement in the T2 stage and had a high PPV (83.3%). However, mild thickening of the MP layer may not only be due to cancer infiltration (T2 stage) but also inflammatory reaction (T1 stage). Moreover, fibrosis and edema that produced hypoechoic changes also made EUS evaluation difficult [26, 27]. For the T4 stage, according to the AJCC 7th/8th edition, tumors involved with the serosa could be categorized as T4, and most primary T3 patients by the AJCC 6th system were placed in the T4 stage. The T4 staging accuracy was highest in pre-operative staging. This result indicates that EUS is an effective method for evaluating serosal invasion, largely because gastric wall outer edge irregularities are a good indicator of cancer invasion (PPV, ~ 93%).

In addition, the tumor location and histological and echoendoscope types are associated with the accuracy of EUS staging; where tumors located in the gastric angulus were an independent indicator associated with EUS overstaging. The EUS accuracy was higher in well-differentiated histological types than in other parts, and tumors in signet ring cell adenocarcinoma were related to EUS understaging. The reason may be that tumors that differentiate into signet ring cell adenocarcinoma are commonly scirrhous and tend to have tumor microinvasion that cannot be detected by EUS [28, 29]. These results suggest that cancers with these features may be more severe than those indicated by EUS pre-operative staging. Careful attention is required during EUS examination to precede the therapeutic schedule for gastric cancer with these characteristics.

The present study has some limitations that require further discussion. First, EUS staging was performed using retrospective still images. Reviewing still images did not provide information about lesion flexibility to predict invasion depth. Second, the sample of patients was relatively small and limited the application of the results. Third, the T stage, including subgroups such as T1a vs. T1b and T4a vs. T4b, could be further discussed. Finally, the EUS accuracies for N/M staging were not compared and should be considered in this study. A multicenter prospective study is required.

## Conclusions

In conclusion, EUS could serve as an accurate technology to determine the infiltration depth of gastric cancers. In view of the T stage, the 6th edition AJCC T staging system may be suited for EUS T staging. The tumor location and histological and echoendoscope types of gastric cancers heavily influenced the EUS staging accuracy. For these patients, it is recommended that gastroenterologists should consider the T stage image characteristics mentioned above.

## Data Availability

The datasets used and/or analyzed during the current study are available from the corresponding author on reasonable request.

## Abbreviations

AJCC: American Joint Committee on Cancer
EUS: Endoscopic Ultrasound
MP: Muscularis propria
TNM: Tumor-lymph node-metastasis
SD: Standard deviation
PPV: Positive predictive value
NPV: Negative predictive value
